# Landscape Dynamics (landDX) an open-access spatial-temporal database for the Kenya-Tanzania borderlands

**DOI:** 10.1038/s41597-021-01100-9

**Published:** 2022-01-18

**Authors:** Peter Tyrrell, Irene Amoke, Koen Betjes, Femke Broekhuis, Robert Buitenwerf, Sarah Carroll, Nathan Hahn, Daniel Haywood, Britt Klaassen, Mette Løvschal, David Macdonald, Karen Maiyo, Hellen Mbithi, Nelson Mwangi, Churchil Ochola, Erick Odire, Victoria Ondrusek, Junior Ratemo, Frank Pope, Samantha Russell, Wilson Sairowua, Kiptoo Sigilai, Jared A. Stabach, Jens-Christian Svenning, Elizabeth Stone, Johan T. du Toit, Guy Western, George Wittemyer, Jake Wall

**Affiliations:** 1 South Rift Association of Land Owners, Nairobi, Kenya; 2 University of Oxford Wildlife Conservation Research Unit, Oxford, UK; 3 University of Nairobi, Department of Geography and Environmental Sciences, Nairobi, Kenya; 4 Kenya Wildlife Trust, P.O. Box 86-00502 Karen, Nairobi, Kenya; 5 Section for Ecoinformatics and Biodiversity, Department of Biology, Aarhus University, Aarhus, Denmark; 6 Center for Biodiversity Dynamics in a Changing World (BIOCHANGE), Department of Biology, Aarhus University, Aarhus, Denmark; 7 Colorado State University, Graduate Degree Program in Ecology, Fort Collins, USA; 8 Ramani Geosystems Ltd, Nairobi, Kenya; 9 Independent (Rijperweg 91, 1462 MD Middenbeemster, The Netherlands; 10 Department of Archaeology and Heritage Studies & IMC, Aarhus University, Aarhus, Denmark; 11 Save the Elephants, Nairobi, Kenya; 12 Mara Elephant Project, Nairobi, Kenya; 13 Smithsonian National Zoo & Conservation Biology Institute, Conservation Ecology Center, Washington, USA; 14 Mammal Research Institute and Department of Zoology & Entomology, University of Pretoria, Pretoria, South Africa; 15 Department of Wildland Resources, Utah State University, Logan, USA; 16 Colorado State University, Dept. of Fish, Wildlife and Conservation Biology, Fort Collins, USA; 17 Wildlife Ecology and Conservation Group, Wageningen University and Research, 6708 PB Wageningen, The Netherlands

**Keywords:** Conservation biology, Developing world

## Abstract

The savannas of the Kenya-Tanzania borderland cover >100,000 km^2^ and is one of the most important regions globally for biodiversity conservation, particularly large mammals. The region also supports >1 million pastoralists and their livestock. In these systems, resources for both large mammals and pastoralists are highly variable in space and time and thus require connected landscapes. However, ongoing fragmentation of (semi-)natural vegetation by smallholder fencing and expansion of agriculture threatens this social-ecological system. Spatial data on fences and agricultural expansion are localized and dispersed among data owners and databases. Here, we synthesized data from several research groups and conservation NGOs and present the first release of the Landscape Dynamics (landDX) spatial-temporal database, covering ~30,000 km^2^ of southern Kenya. The data includes 31,000 livestock enclosures, nearly 40,000 kilometres of fencing, and 1,500 km^2^ of agricultural land. We provide caveats and interpretation of the different methodologies used. These data are useful to answer fundamental ecological questions, to quantify the rate of change of ecosystem function and wildlife populations, for conservation and livestock management, and for local and governmental spatial planning.

## Abstract (summary)

**Table:** 

Measurement(s)	livestock enclosures • agriculture • fence
Technology Type(s)	digital curation
Sample Characteristic - Environment	savanna
Sample Characteristic - Location	East Africa

Machine-accessible metadata file describing the reported data: 10.6084/m9.figshare.16828204

## Background & Summary

**Fig. 1:**
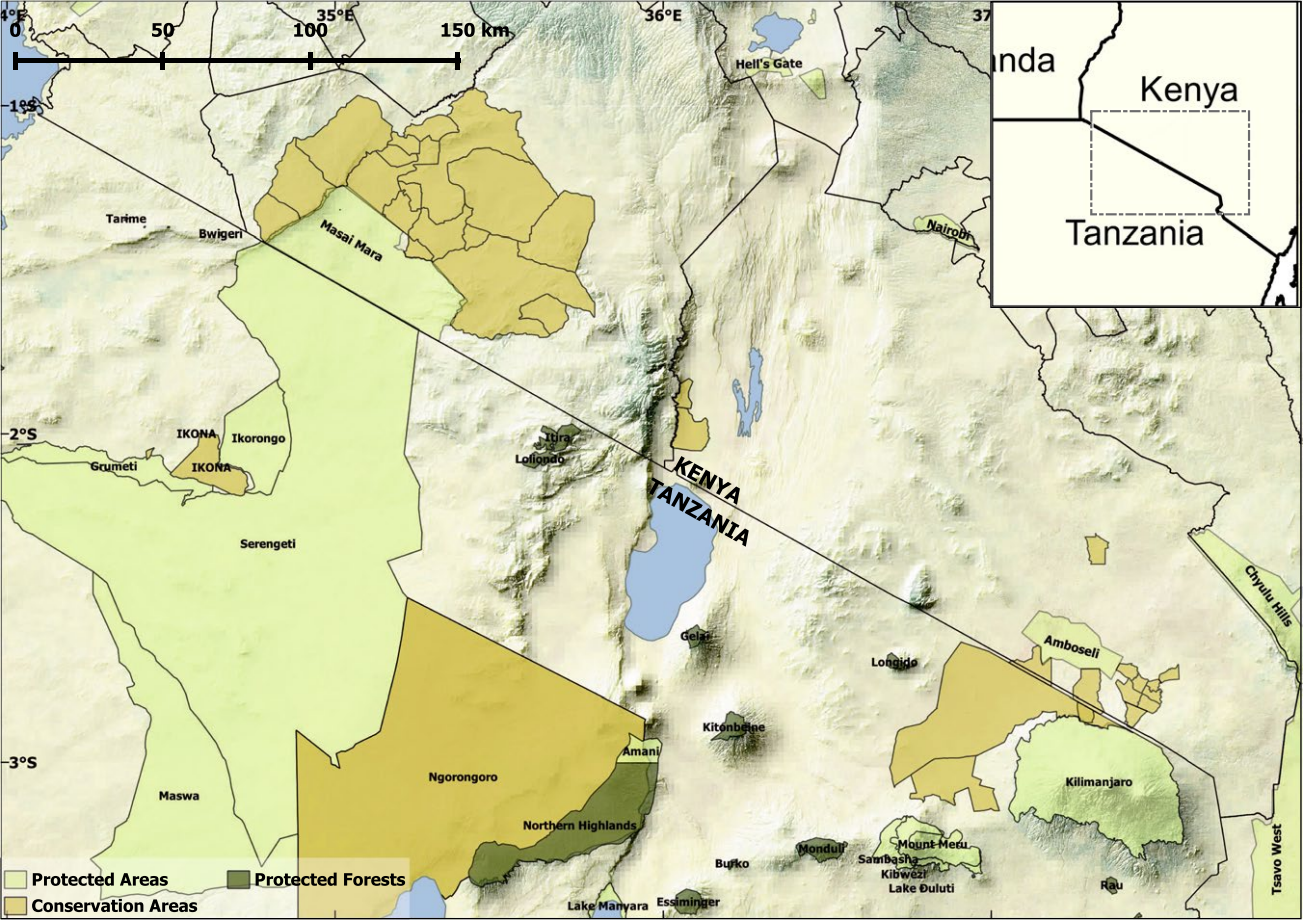
The Kenyan-Tanzania borderland area (roughly 100,000 km^2^), is the focus of this data release. Our initial data-set covers most of Kajiado and Narok counties in Kenya. This region is one of the most important areas globally for conservation. The area consists of 16 national parks and reserves (light green), protected forests, including forest reserves and community protected forests (dark green) and many conservancies, wildlife management areas, game management areas, and conservation areas (orange).

The Kenya – Tanzania borderlands cover roughly 100,000 km^2^ spanning the rangelands between southern Kenya and northern Tanzania (Fig. 1). This region is one of the most biodiverse in the world and contains some of Earth’s highest densities of large mammals^1,2^. Because of this, and the value they generate through tourism, the region also has some 16 national parks and forest reserves stretching from the Serengeti and Maasai Mara in the west to Tsavo and Mkomazi in the east. Adjacent and between these government-protected areas are scores of non-government run conservancies, wildlife management areas, wildlife ranches, and game controlled areas. Despite the importance of this region for wildlife conservation, the borderlands are dominated by livestock^3^. This region is predominantly home to the Maasai people, who are traditionally transhumant or seasonally moving pastoralists^4^. Maasai herding practices and relative tolerance towards wildlife have allowed for continued cohabitation of wildlife and people^2^.

However, extensive pastoralism and wildlife are under threat, particularly on the Kenyan side of the border. Both suffer from the negative effects of fragmentation^5^ driven by complex processes including policy change, economic drivers, and globalization, which have encouraged and driven subdivision and fencing, and the expansion of subsistence and mechanized agriculture^6–8^. This has halted migrations of wildlife and livestock, leading to wide-scale decreases in wildlife numbers^7,9–11^, decreases in connectivity between populations and increasing genetic isolation^12^, and losses in rangeland productivity and heterogeneity^13^. Additionally, many areas have experienced the loss of traditional grazing management structures, including increasing rates of sedentarisation (permanent settlement of transhumant pastoralists), causing rangeland degradation^14^.

There have been many attempts to map these changes, including mapping of fencing, livestock enclosures (bomas), wildlife numbers, agricultural expansion, and habitat change^7,8,15–18^. These data are useful for a range of applications: to answer fundamental ecological questions, to quantify the rate of change of ecosystem function, pastoralists strategies, and wildlife populations - especially wildlife mobility, for conservation planning and mitigating threats to wildlife, for livestock management and planning, and local and governmental spatial planning. However, much of these data have remained inaccessible, disparate, or at smaller spatial or temporal scales than required to address regional challenges. Across the region, there have been calls to increase data sharing and collaborations between data owners, involving initiatives such as the Borderlands Conservation Initiative, SOKNOT WWF, One Mara Research Hub, and Amboseli-Tsavo-Kilimanjaro Spatial Data Infrastructure (ATK-SDI).

Here we present a first step towards providing a regional scale open-access spatial (and temporal) database, through a permanent and updatable web server for the Kenya-Tanzania borderlands. The authors have initially compiled data for three of the most important anthropogenic structures covering an area of over 30,000 km^2^, focusing on the rangelands of southern Kenya the extent of fencing across the region, the distribution of livestock enclosures (*enkangs* or bomas), and the extent of agricultural land use. Unlike previous efforts to collate this type of data, we have now synthesized data at a scale large enough to be relevant for addressing and understanding the fragmentation in the region. The data from this study are accessible through the Landscape Dynamics (landDX) database hosted by the Mara Elephant Project (MEP). The landDX database is based on the ArcGIS online data infrastructure (https://www.arcgis.com/home/search.html?q=landDx), where it will be updated as and when new imagery, data, and project funding become available. A static version of this data is also made available through the Oxford Research Archive.

## Methods

### Data collection

**Fig. 2:**
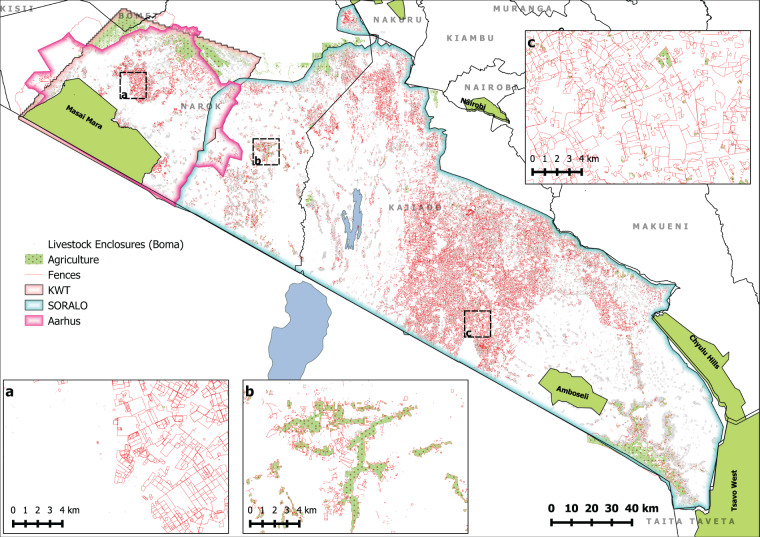
A map of the dataset presented in the first release of this database. Including the rough area covered by each data-collection method (no area is given for MEP as data collection is ad-hoc), and the three datasets released livestock enclosures (bomas), agricultural land use, and fencing.

Data were collected from a variety of partners and sources across the region: South Rift Association of Land Owners, Aarhus University, Kenya Wildlife Trust, and the Mara Elephant Project. The study area covers the majority of the Kenyan counties of Kajiado and Narok (Fig. 2). The data extend into parts of Machakos and Nakuru counties, and the dataset is clipped to the Siria Escarpment and Nyakweri forest to the west, and the extensive farms along the Mau Escarpment to the north (Fig. 2). Beyond these limits, the land use is predominantly agricultural.

### Aarhus university

Løvschal *et al*., 2017 mapped the changes in fences located within the Greater Mara (5,771 km^2^), using Landsat satellite images between 1985 and 2016 with a resolution of 30 m (Table 1; Fig. 2). In this study, only Landsat satellite images with similar optical sensors and spatial resolution were used, and images with a cloud cover of <1% were retained. All images were from mid-January to late-February to minimize the effect of changing seasons. The digitization of fences was done manually (by a single individual) based on visual interpretation using ArcMap 10.3.1, by looking at characteristic homogeneous areas using three different colour composites as well as a version based on normalized difference vegetation index (NDVI). When an assessment of one year was conducted, it was compared to the mapped fences from previous years to ensure that fences from previous years would not be overlooked due to difficulties in detecting them in later years.

**Table 1:** Metadata for the data currently publicly available from the data portal.

Data Type	Data Geometry Type	Data Contributor	Total Area covered	Number of objects/size	Methods	Projection
Livestock Enclosures (Boma)	Point	SORALO	25,041 km^2^	26,309	Google Earth Digitisation	WGS84
		KWT	6,373 km^2^	4,714	Google Earth and Bing maps Digitization	
		*Total*	*31,414 km*^2^	*31,0*2*3*		
	Polygon	SORALO	25,041 km^2^	32,592	Google Earth Digitisation	WGS84
		KWT	6,373 km^2^	5,118	Google Earth and Bing maps Digitization	
		*Total*	*31,414 km*^2^	*37,710*		
Fences	Polylines	SORALO	25,041 km^2^	37,847.5 km	Google Earth Digitisation	WGS84
		Aarhus University	5,771 km^2^	2,104.7 km (2016 data)	Digitisation of Landsat Imagery	
		Mara Elephant Project		1,787 km	Ground collection	
		*Total*	*30,5*2*7 km*^2^	41,739.2 km		
Agriculture	Polygon	SORALO	25,379 km^2^	785.9 km^2^	Google Earth Digitisation	WGS84
		KWT	6,373 km^2^	676.6 km	Google Earth and Bing maps Digitization	
		*Total*	*31,414 km*^*2*^	*1,462.5 km*^*2*^		

### South rift association of land owners data (SORALO)

In 2019 and 2020, SORALO collected data for the entirety of Kajiado county, parts of Machakos and Nakuru county, and a considerable portion of east Narok county, totalling 25,000 km^2^ (Table 1; Fig. 2). Using QGIS^19^, a Google Earth layer was used to digitize bomas, visible fence lines, and agricultural land use using a 1 × 1 km search grid. These data were digitized in collaboration with Ramani Geosystems Ltd. (www.ramani.co.ke).

**Fig. 3:**
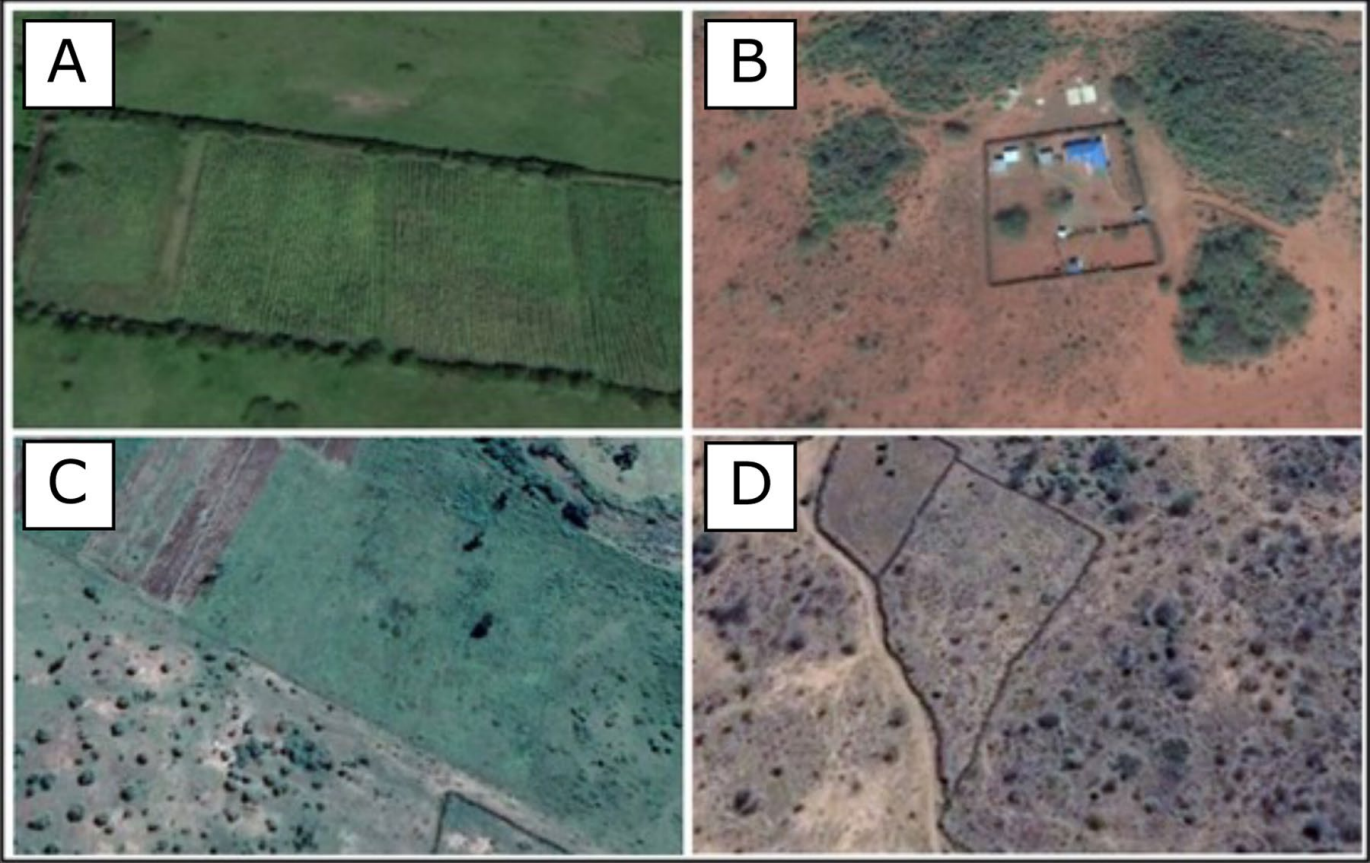
Example of the fence lines that are visible with Google Maps imagery and which were digitized by SORALO.

**Fig. 4:**
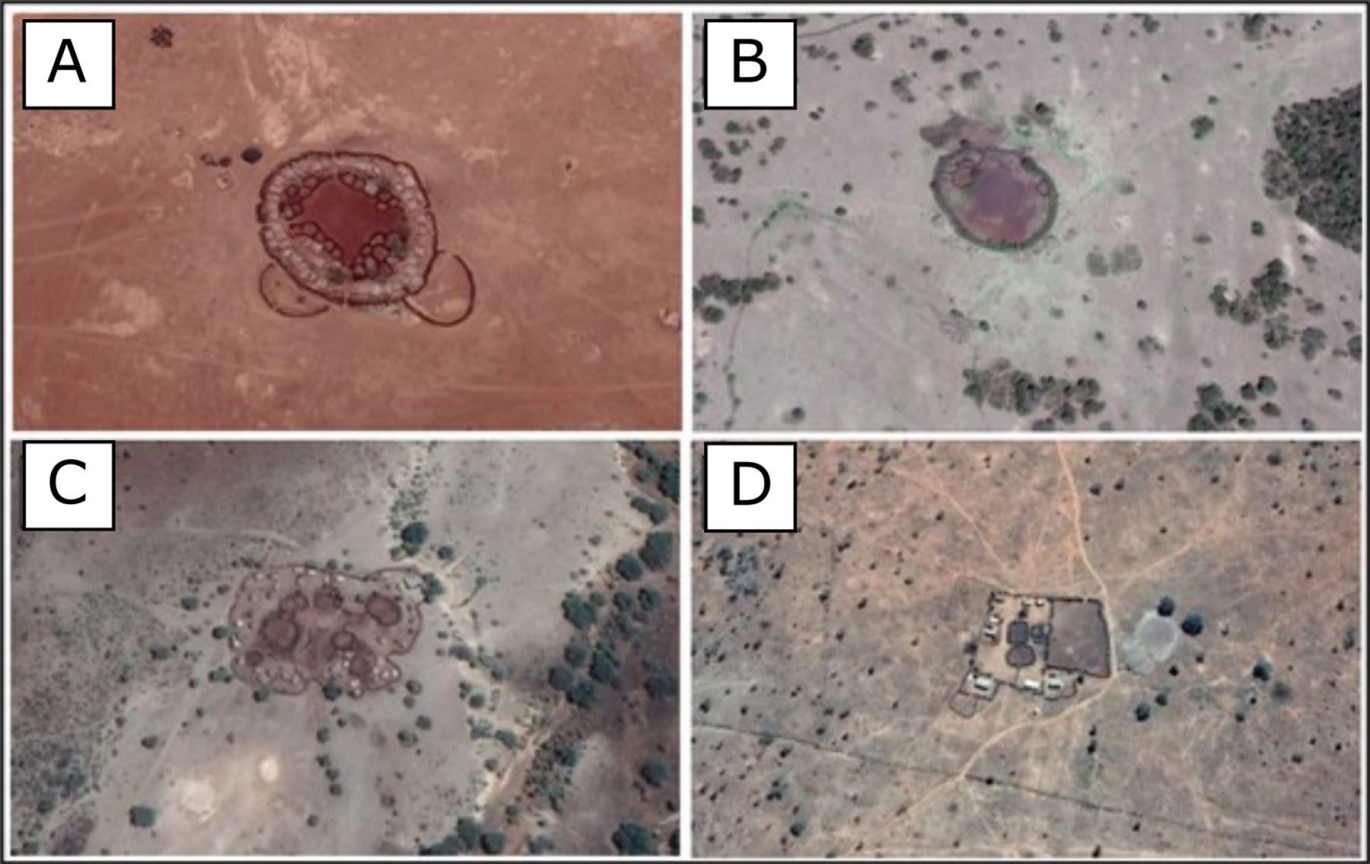
Example of the different livestock enclosure types (bomas) that are visible with Google Earth and Bing maps imagery and which were digitized by SORALO and KWT. Note the visible change in terrain colour and texture where livestock is held and the surrounding brush fences.

Brush fences are easily distinguishable from Google Earth imagery due to their width and dark brown colour (Fig. 3). These types of fences represent the majority of fencelines present in this semi-arid region. However, this imagery alone is not accurate enough to identify the thin wire fences more common to agricultural land. Instead, an abrupt change in land use, especially if it is in a straight line, likely indicates a wire fence. In this manner, digitization of these types of fence lines used a similar method to the Aarhus University team, searching for homogenous areas of vegetation, urban development, or agriculture, distinct from the surrounding vegetation, which must have resulted as a consequence of fencing (Fig. 3). We converted these digitized fence lines into polygons and manually searched through them to identify areas of agricultural production identifiable from the Google Earth imagery. Livestock enclosures (bomas) vary across the region, ranging from permanent settlements to more traditional temporary and permanent bush enclosures (Fig. 4). We therefore only digitized areas which were clearly used to hold livestock at the date the satellite image was collected, rather than the full extent of a settlement. This is clear from the distinctive colour of manure and a continuous fenced perimeter delimiting the boma.

A date layer was generated from Google Earth imagery by manually digitizing the underlying imagery data boundaries for the study region, and the corresponding imagery dates spatially joined to the fencing data (See Validation).

### Kenya wildlife trust (KWT)

Klaassen and Broekhuis (2018)^18^ collected data on the human footprint in the Maasai Mara ecosystem by manually digitizing all anthropogenic structures using QGIS in a similar way to SORALO. We created a 1 × 1 km grid layer covering over 6,000 km^2^ (Table 1; Fig. 2), which included the Maasai Mara National Reserve, the surrounding conservancies and community land. Within this grid layer, we documented all human settlements using the OpenLayers for both Google Earth and Bing maps. The human settlements included agricultural land, settlements, livestock enclosures (bomas), settlements with adjacent livestock enclosures, towns, dams, tourist camps, and other and unknown developments. Here, we present the livestock enclosures (boma) data, settlements with adjacent livestock enclosures, and agricultural land. No date attribute is provided with this layer, although all data were collected using imagery that was available up to 2017.

### Mara elephant project (MEP)

Beginning in November 2019, MEP identified fences on the ground using a custom mobile mapping application ‘*TerraChart’* based on the Esri Android SDK (https://play.google.com/store/apps/details?id=org.maraelephantproject.terrachart). Three field assistants using motorbikes have mapped 1787 km of fencing as of November 2020 (Table 1). Fences are classified by type (Electric, Wire, Stone, Wood post, Cactus, Other) and based on strand count for wire/electric fences. Data collection is ongoing using other datasets (see Table 1) to target ground collection.

### Data amalgamation

All data were amalgamated and processed using R^20^ and the *sf* package^21^.

- **Agricultural data:** we combined the agricultural polygon data from the KWT and SORALO.
- **Fence Polylines:** we combined the fenceline data from SORALO and Aarhus university into a single database. We used the 2016 Aarhus data, the MEP data, and the SORALO fencing data to present our best estimate of the current extent of fencing across the region. The spatial-temporal nature of the dataset will allow for future comparison of the Aarhus data across years (1985–2020). The Aarhus data were converted from a polygon to a polyline to match the format of the SORALO data.
- **Boma Polygons:** we combined the boma polygon data from the KWT and SORALO.
- **Boma Points:** we calculated the centroids of the boma polygon data from KWT and SORALO. In some bomas, there are multiple holding areas for livestock (Fig. 4C). To produce a single point for each boma we create a buffer of 12.5 m around each polygon and unioned any overlapping polygons before finding the polygon’s centroid.

## Data Records

**Fig. 5:**
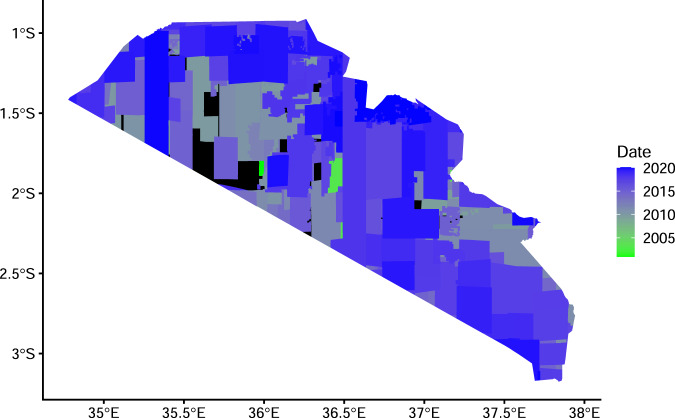
The date of acquisition of satellite imagery from Google Earth used in the SORALO mapping process. Black polygons have no date attribute.

We provide an initial four types of data that extend across southern Kenya (Fig. 5): fences demarcated as polylines, polygons of livestock enclosures, centroids of points from the boma polygons layer, and agricultural polygons. These data are accessible from the Landscape Dynamics data portal (https://www.arcgis.com/home/search.html?q=landDx_export). This data portal is hosted on Esri ArcGIS Online and provides a web map service in addition to available data which will be updated over time. In addition, data are available through the Oxford University Research Archive (10.5287/bodleian:qqv4EdRnQ)^22^, however it will not be updated.

These data are stored as three feature collections on ArcGIS online, dependent on the geometry type of the data: polylines; polygons; or points. A Python script is run whenever the database has been updated from a Jupyter notebook on ArcGIS online, to produce a timestamped set of ESRI shapefiles and File Geodatabases from these feature collections. Publicly available data are searchable through the ArcGIS Online website. We provide two shapefiles which contain only active data (see below), and both active and inactive data.

Each geometry feature type (point, polyline, polygon) share a standardized data table format:

1. *type*: the type of feature represented by the geometry (e.g. Fence_Other or Fence_Electric_Unknown)
2. *name*: if available the name of the feature
3. *short_name:* if available the shortened name of the feature
4. *active:* a binary field indicating whether the data should be considered currently relevant for the landscape (1)
5. *collect_user:* the person or organization responsible for the data collected
6. *collect_method:* the methodology used for data collection (such as Google Earth Digitisation, Landsat Digitisation, or GPS)
7. *collect_date:* when known the date of the data acquisition, including the date of imagery used during digitization or field data collection (UTC)
8. *ground_verified:* indicates whether the data has been confirmed on the ground.
9. *spatial_feature_owners:* the owner of the spatial feature
10. *spatial_data_owners:* the owner of the spatial data
11. *data_availabiltiy: an* indicator of the open-access format
12. *other_id*: attribute data passed in JSON format containing information specific to the data contributor
13. *attributes*: a javascript object notation (JSON) column to store additional attribute, type or labels for a given feature
14. *globalid*: a globally unique identifier (guid) value for the feature
15. *parent_guid*: This describes a parent-child relationship between different features (even across geometry types) and allows the comparability of the connected features mapped across different years but whose geometry and attributes might vary.

Table 1 provides the details of the public data records released with this data descriptor. This includes the data geometry type, format, the data source, the total area covered (linked to the area of interest files), number and/size of the objects in the method used to collect the data, and the projection of the data.

### Overview of data

Figure 2 provides an overview of the geographical location of the data sources collected, contained in the area of interest shapefile. In total, we collected data from over 31,000 km^2^. In total our database consists of 37,710 livestock enclosure polygons; 31,023 livestock enclosure points; 41,739 km of fencing; and 1,465.5 km^2^ of agricultural areas.

## Technical Validation

Aarhus University, SORALO and KWT digitized bomas, fences and agriculture in a systematic manner using available satellite imagery (see methods). All digitization was re-checked by supervisors, to ensure that no data had been missed, and was adjusted following quality control where and when required. All data were then manually checked by conservation practitioners knowledgeable of the study area. Both the spatial resolution and temporal sampling of the data may present limitations to its accuracy and usage.

### Spatial resolution

For both the KWT and SORALO datasets collected using Google Earth, we used the latest Google Earth imagery. Additionally, for KWT’s dataset, we also used the latest Bing maps imagery. However, the spatial resolution of this Google Earth and Bing maps data varies. Resolution can be as high as ~0.5 m, while a few remaining areas still rely on Landsat Imagery with a resolution of 30 m. However, the quality of the Google Earth and Bing maps imagery was generally high enough across the study area to accurately delineate bomas, fencelines and agricultural land. Figures 3 and 4 provide examples of areas that would be digitized, with the boundaries of the boma and fence lines clearly visible.

The fencing data collected by Aarhus University used Landsat Imagery at 30 m resolution and smaller fences may be missing from the dataset as they are harder to distinguish. This is also true for wire fence (the predominant type of fencing around the Maasai Mara; Fig. 3C). Vegetation differences used to identify these fence lines may take some time to develop. Therefore, there may be an underestimate of the fences mapped, especially in those regions with high usage of wire fences.

It must be noted that images from Google Earth have an overall positional root mean squared error of 39.7 m, which may impact the interpretation of this dataset^23^. We believe that these errors are acceptable for our first attempt at collecting landscape-scale data, and will be refined over time with improved imagery and ground-truthing. Landsat data has a root mean squared error usually below the size of a pixel, with 90% of pixels having less than 12 m deviation (^1^ https://www.usgs.gov/media/videos/landsat-collections-rmse).

### Temporal variation

The most likely discrepancies in data quality will arise from temporal variation in fencing placement, boma usage and placement, and agricultural change. Google Earth data were used for SORALO, using data available up to February 2020. Google Earth and Bing maps data were used for KWT, with data up to 2017. The weighted mean imagery date for SORALO (weighted by the area covered) was the 9th of September 2016 and ranged from 15th of December 2000 to 12th of February 2020 (Fig. 5). Where possible we have added a date-time stamp to the boma, agriculture and fencing dataset to best match the date the satellite imagery was acquired, or when it was collected on the ground. However, KWT and some SORALO data lack date attribute, the latter because no date stamp was found in Google Earth, and the former because no date was recorded for any data. The Aarhus University fencing data are from a Landsat Image from January 2016, and the MEP data are from on-the-ground collection. Our database is built so that as new or updated data become available, from both new satellite imagery and ground-based identification, the data layer can be adjusted (see below).

### Livestock enclosure validation

We used data on the location of SORALO livestock enclosures from the Magadi region^24^ (collected using handheld GPS devices), to estimate the accuracy of our data collection. The SORALO ground-truthed database contains 668 bomas, which have been occupied at least once during 2014–2017. In the same area, our boma points database contains 573 bomas (85%) of which 41.2% (n = 275) are within 100 m of ground-truthed points and 87.7% (n = 586) are within 500 m of the ground-truthed points. These ground-truthed points may have inaccuracies from their data collection. Also, many livestock enclosures distant from ground-truthed points are newer than the ground-truthing dataset.

### Agricultural land validation

We compared our agricultural data layer to a commonly used global open source data layer, the 2015 GFSAD30AFCE 30-m for Africa: Cropland Extent Product (www.croplands.org). Our layer agreed with the Cropland Extent Product across 856 km^2^ of cropland. However, our layer demarcated 455 km^2^ (34.4% of the total extent) more agricultural land than was found in the 30 m Cropland Extent Product, because many small areas of subsistence farming had not been detected by this global layer. Additionally, the Cropland Extent Product contained 468 km^2^ (35.3% of the Cropland Extent Product) of agricultural extent not captured in our layer. Much of this was on the periphery of large continuous agricultural areas and appears inaccurately mapped by the global product.

### Continual validation and improvement of database

Ongoing ground-truthing exercises by the Mara Elephant Project and other partners will improve the quality of the database over time, particularly the datasets on wire fencing in the Mara region. To do so the *TerraChart app* combined with a QuickCapture app (to validate fence lines and boma locations using aerial reconnaissance) are integrated into the ArcGIS online framework, and following validation both manually and using automated Python script, can be used to update the features collection database.

Additionally, any data currently held in the private domain can be easily integrated into this database, and made available to the public domain with approval. Linking these features using a parent ID allows for not only the addition of new features, but improved spatial accuracy of old features, and temporal changes to features to be captured.

This database will be continually improved over time. For example, current efforts from conservation partners in the region have resulted in large scale acquisition of high resolution, up-to-date, satellite imagery which will be further used to refine this database.

## Usage Notes

We aim to provide a comprehensive layer of the most recent anthropogenic impacts for use at regional scales. These data, however, still require the end-user to interpret any results based on the method used to collect it, as well as the temporal dynamics of each data type. This is particularly relevant for areas under change where fencing is rapidly increasing. Temporal aspects of the data are also important for interpreting the occupancy of livestock enclosures (bomas) which are likely to change seasonally as forage and water distribution change, but also over longer periods as socio-economic pressures alter livestock management and governance practices (see validation above). Data collected through digitization of Google Earth and Landsat imagery may likely underestimate the extent of fencing where wire, rather than brush fencing is used, and where visible changes in vegetation condition are not apparent. Continual field-based data collection from the MEP team, and other project partners, will improve the accuracy of the digitization products. In addition, some datasets may overlap in the public release, including the Aarhus and MEP data. We provide all datasets in one release so that the end-user can make their own decision on filtering and combing data, based on their requirements. The area of each feature class (points, polygons, and lines) is provided for this purpose within the downloadable products.

This database will be updated and continually validated as new imagery, external data sources, and ground-truthed data are collected. The end-user should note this and expect changes to the database over-time. This includes the addition of other data sources such as land-use and land-cover maps, protected area boundaries, rivers, and roads. For example, already 3828 km of roads in the Mara region have been mapped. These data, and updates, will be made available by download through ArcGIS online.

While other options exist to host open access data, such as Open Street Map (OSM), we chose to create our database and host it through ArcGIS online for several reasons. First, we want to capture the temporal dynamics of change within this region, which we are unable to do in OSM. Second, we want to have control over the contributions into this database so that only verified data, matching our required gaps in the database, are added. Third, it allows contributors to share data under varying degrees of access, dependent on the data’s sensitivity and ownership rights. Finally, our database allowed meta-data specific to the datasets we are collecting, as opposed to conforming to other standard tags.

Data owners who are interested in contributing open-access data to this database are welcome to reach out to the lead author. Our initial data release provides some of the most important features for determining the level of fragmentation and losses of mobility in the region. Over time, we hope to grow the geographic range of the database and the data sources. We anticipate that this initiative will encourage data-sharing in the Kenya-Tanzania borderlands area (and more widely in East Africa, with this approach already being adopted in northern Kenya) and provide easy access to updatable data sources, which are critical to the conservation, management, and understanding of this region. In addition, we hope the landDX database structure encourages more frequent and better standards of data sharing in the broader conservation and landscape management communities.

## Data Availability

No custom code was used to generate the data.
